# Activity Recognition Invariant to Wearable Sensor Unit Orientation Using Differential Rotational Transformations Represented by Quaternions

**DOI:** 10.3390/s18082725

**Published:** 2018-08-19

**Authors:** Aras Yurtman, Billur Barshan, Barış Fidan

**Affiliations:** 1 Department of Electrical and Electronics Engineering, Bilkent University, Bilkent, Ankara 06800, Turkey; yurtman@ee.bilkent.edu.tr; 2 Department of Mechanical and Mechatronics Engineering, University of Waterloo, 200 University Ave W, Waterloo, ON N2L 3G1, Canada; fidan@uwaterloo.ca

**Keywords:** activity recognition and monitoring, patient health and state monitoring, wearable sensing, orientation-invariant sensing, motion sensors, accelerometer, gyroscope, magnetometer, pattern classification

## Abstract

Wearable motion sensors are assumed to be correctly positioned and oriented in most of the existing studies. However, generic wireless sensor units, patient health and state monitoring sensors, and smart phones and watches that contain sensors can be differently oriented on the body. The vast majority of the existing algorithms are not robust against placing the sensor units at variable orientations. We propose a method that transforms the recorded motion sensor sequences invariantly to sensor unit orientation. The method is based on estimating the sensor unit orientation and representing the sensor data with respect to the Earth frame. We also calculate the sensor rotations between consecutive time samples and represent them by quaternions in the Earth frame. We incorporate our method in the pre-processing stage of the standard activity recognition scheme and provide a comparative evaluation with the existing methods based on seven state-of-the-art classifiers and a publicly available dataset. The standard system with fixed sensor unit orientations cannot handle incorrectly oriented sensors, resulting in an average accuracy reduction of 31.8%. Our method results in an accuracy drop of only 4.7% on average compared to the standard system, outperforming the existing approaches that cause an accuracy degradation between 8.4 and 18.8%. We also consider stationary and non-stationary activities separately and evaluate the performance of each method for these two groups of activities. All of the methods perform significantly better in distinguishing non-stationary activities, our method resulting in an accuracy drop of 2.1% in this case. Our method clearly surpasses the remaining methods in classifying stationary activities where some of the methods noticeably fail. The proposed method is applicable to a wide range of wearable systems to make them robust against variable sensor unit orientations by transforming the sensor data at the pre-processing stage.

## 1. Introduction

As a consequence of the development and pervasiveness of sensor technology and wireless communications, wearable sensors have been reduced in size, weight, and cost, gained wireless transmission capabilities, and been integrated into mobile devices such as smart phones, watches, and bracelets [1]. Such smart devices, however, have limited resources. Their effectiveness is determined by the screen size, sensor, computing processor, battery and storage capacities, as well as the wireless data transmission capability [2,3]. Activity recognition with wearables has various potential applications in the healthcare domain in the form of medical state monitoring, memory enhancement, medical data access, and emergency communications [4,5]. Health state monitoring and activity recognition using wearable sensors is advantageous compared to approaches based on computer vision and radio frequency identification that rely on external sensors such as cameras or antennas [6].

**Figure 1:**
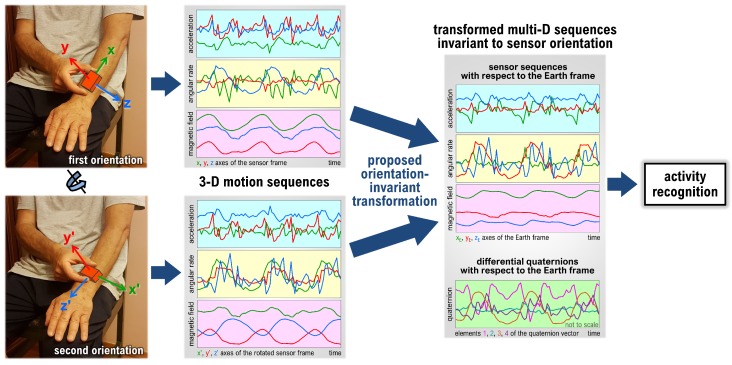
An overview of the proposed method for sensor unit orientation invariance.

With the advancements mentioned above, placing wearable devices on the body properly has become a challenging and intrusive task for the user, making wearable devices prone to be fixed to the body at incorrect orientations. For instance, disabled, injured, elderly people or children whose health, state, or activities can be monitored using wearables [7] tend to place the sensor units at incorrect or variable orientations. Mobile phones can be carried in pockets at different orientations. However, the majority of existing wearable activity recognition studies neglect this issue and assume that the sensor units are properly oriented or, alternatively, use simple features (such as the vector norms) that are invariant to sensor unit orientation. In this study, we focus on orientation invariance in a generic activity recognition framework. Our aim is to develop a methodology that can be applied at the pre-processing stage of activity recognition to make this process robust to variable sensor unit orientation, as depicted in Figure 1.

We utilize tri-axial wearable motion sensors (accelerometer, gyroscope, and magnetometers) to capture the body motions. Data acquired by these sensors not only contain information about the body movements but also about the orientation of the sensor unit. However, these two types of information are coupled in the sensory data and it is not straightforward to decouple them. More specifically, a tri-axial accelerometer captures the vector sum of the gravity vector and the acceleration resulting from the motion. A tri-axial gyroscope detects the angular rate about each axis of sensitivity and can provide the angular velocity vector. A tri-axial magnetometer captures the vector sum of the magnetic field of the Earth and external magnetic sources, if any.

**Figure 2:**
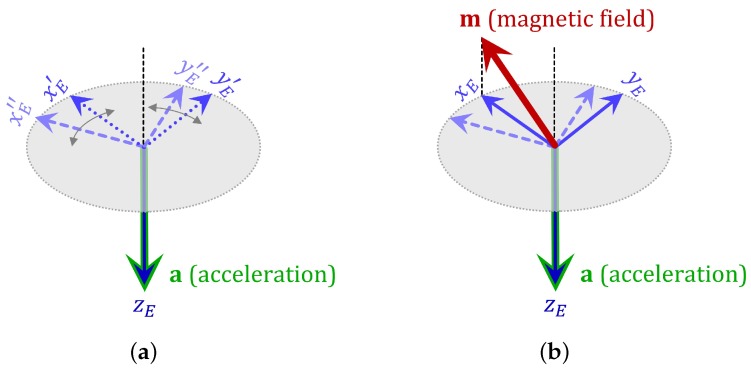
(**a**) With only the acquired acceleration field vector **a**, there exist infinitely many solutions to the sensor unit orientation (two are shown); (**b**) the acquired magnetic field vector **m** uniquely identifies the sensor unit orientation.

The acceleration vector acquired by an accelerometer approximately points in the down direction of the Earth frame, provided that the gravitational component of the total acceleration is dominant over the acceleration components resulting from the motion of the sensor unit. However, even if the acceleration vector consists of mainly the gravitational component, by itself it is not sufficient to estimate the sensor unit orientation because there exist infinitely many solutions to the sensor unit orientation, obtained by rotating the correct solution about the direction of the acquired acceleration vector (Figure 2a). Hence, we need to incorporate the magnetometer into the orientation estimation as well. The magnetic field vector acquired by a magnetometer points in a fixed direction in the Earth frame (the magnetic North) (Figure 2b), provided that there are no external magnetic sources or distortion and the variation of the Earth’s magnetic field is neglected.

By taking the reference directions obtained from the accelerometer and the magnetometer as the vertical axis and the (magnetic) North axis of the Earth frame, respectively, we can calculate the orientation of the sensor unit with respect to the Earth frame. However, this estimation is reliable only in the long term because the gravity component is superposed with the acceleration caused by the motion of the unit and the Earth’s magnetic field is superposed with the external magnetic sources (if any). Hence, we also estimate the sensor unit orientation by integrating the gyroscope angular rate output, which is reliable only in the short term because of the drift error [8]. To obtain an accurate orientation estimate both in the short and long term, we merge these two sources of information. Thus, we exploit the information provided by the three types of sensors to determine the sensor unit orientation with respect to the Earth frame as a function of time.

Once we estimate the sensor unit orientation with respect to the Earth frame, we can transform the acquired data from the sensor frame to the Earth frame such that they become invariant to sensor unit orientation. In addition, to include the information about the rotational motion of the sensor unit, we represent the sensor unit rotation between consecutive time samples in the Earth frame by using a similarity transformation. We show that appending this rotational motion data to the sensor data and representing both in the Earth frame improves the activity recognition accuracy. Figure 1 provides an overview of the proposed method with experimentally acquired sensor sequences during a walking activity.

We utilize widely available sensor types and do not make any assumptions about the sensor configuration, data acquisition, activities, and activity recognition procedure. Our proposed method can be integrated into existing activity recognition systems by applying a transformation to the time-domain data in the pre-processing stage without modifying the rest of the system or the methodology. We outperform the existing methods for orientation invariance and achieve an accuracy close to the fixed orientation case.

The rest of the article is organized as follows: In Section 2, we summarize the related work on wearable sensing that allows versatility in sensor placement. Section 3 presents the transformations applied to the sensor data to achieve orientation invariance. In Section 4, we describe the dataset together with the proposed and existing methodology on orientation invariance, explain the activity recognition procedure, and present the data analysis results including accuracies and run times of the data transformation techniques and the classifiers. In Section 5, we provide a discussion of the results. Section 6 summarizes our contributions, draws conclusions, and indicates some directions for future research.

## 2. Related Work

Although most of the existing activity recognition studies assume fixed sensor unit orientations [9,10], a number of methods have been proposed to achieve orientation invariance with wearable sensors. These methods can be grouped as transformation-based geometric methods, learning-based methods, and other approaches.

### 2.1. Transformation-Based Geometric Methods

A straightforward method for achieving orientation invariance is to calculate the magnitudes (the Euclidean norms) of the 3D vectors acquired by tri-axial sensors and to use these magnitudes as features in the classification process instead of individual vector components. When the sensor unit is placed at a different orientation, the magnitude of the sensor readings remains the same, making this method invariant to sensor unit orientation [10,11,12]. Reference [10] states that a significant amount of information is lost with this approach and the accuracy drops off even for classifying simple daily activities. Instead of using only the magnitude, references [13,14,15] append the magnitude of the tri-axial acceleration vector as a fourth axis to the tri-axial data. Reference [13] shows that this modification slightly increases the accuracy compared to using only the tri-axial acceleration components. Even if the magnitude of the acceleration is not appended to the data, the limited number of sensor unit orientations considered (only four) allows accurate classification to be achieved with Support Vector Machine (SVM) classifiers [13]. Reference [16] uses the magnitude, the *y*-axis data, and the squared sum of *x* and *y* axes of the tri-axial acceleration sequences acquired by a mobile phone, assuming that the orientation of the phone carried in a pocket has natural limitations: the screen of the phone either faces inward or outward.

In a number of studies [17,18,19], the direction of the gravity vector is estimated by averaging the acceleration vectors in the long term. This is based on the assumption that the acceleration component associated with daily activities averages out to zero, causing the gravity component to remain dominant. Then, the amplitude of the acceleration along the gravity vector direction and the magnitude of the acceleration perpendicular to that direction are used for activity recognition [17,18,19], which is equivalent to transforming tri-axial sensor sequences into bi-axial ones. In terms of activity recognition accuracy, in reference [17], this method is shown to perform slightly better and in reference [19], significantly worse than using only the magnitude of the acceleration vector.

In addition to the direction of the gravity vector, reference [20] also estimates the direction of the forward-backward (saggital) axis of the human body based on the assumption that most of the body movements as well as the variance of the acceleration sequences are in this direction. The sensor data are transformed into the body frame whose axes point in the direction of the gravity vector, the forward-backward direction of the body that is perpendicular to that, and a third direction perpendicular to both, forming a right-handed coordinate frame. The method in [20] does not distinguish between the forward and backward directions of the body, whereas reference [10] determines the forward direction from the sign of the integral of the acceleration as the subject walks.

Reference [21] assumes that incorrect placement of a sensor unit causes only shifts in the class means in the feature space. The class means of a Bayesian classifier are adapted to the data by using the expectation-maximization algorithm, and it is shown that the accuracy improves for one dataset and diminishes for another. To test for orientation invariance, sensor data are artificially rotated either about the *x* or *z* axis of the sensor unit. In this study, sensor unit rotation about an arbitrary axis is not considered and the assumption regarding the shifts in the class means is a strong one since such shifts may not always be significant.

Reference [22] proposes a coordinate transformation from the sensor frame to the Earth frame to achieve orientation invariance. To transform the data, the orientation of a mobile phone is estimated based on the data acquired from the accelerometer, gyroscope, and magnetometer of the sensor unit embedded in the device. An accuracy level close to the fixed orientation case is obtained by representing the sensor data with respect to the Earth frame. However, only two different orientations of the phone are considered, which is a major limitation of the study in [22]. Reference [23] calculates three principal axes based on acceleration and angular rate sequences by using principal component analysis (PCA) and represents the sensor data with respect to these axes.

### 2.2. Learning-Based Methods

Reference [24] proposes a high-level machine-learning approach for activity recognition that can tolerate incorrect placement (both position and orientation) of *some of* multiple wearable sensor units. In the standard approach, features extracted from all the sensor units are aggregated and the activity is classified at once. In reference [24], the performed activity is classified by processing the data acquired from each sensor unit separately and the decisions are fused by using the confidence values. The proposed method is compared with the standard approach for different sets of activities, features, and different numbers of incorrectly placed sensor units by using three types of classifiers. When the subjects are requested to place the sensor units at any position and orientation on the appropriate body parts, incorrect placement of some of the units can be tolerated when all nine units are employed, but not with only a single unit.

Among the references [25,26,27] that employ deep learning for activity recognition, reference [27] increases robustness to variable sensor unit orientations by summing the features extracted from the x,y,z axes.

### 2.3. Other Approaches

Reference [28] proposes to classify the sensor unit orientation to compensate for variations in orientation. Dynamic portions of the sensor sequences are extracted by thresholding the standard deviation of the acceleration sequence and four pre-determined sensor unit orientations are perfectly recognized by a one-nearest-neighbor (1-NN) classifier. Then, the sensory data are rotated accordingly prior to activity recognition. However, the number of sensor unit orientations considered is again very limited and the direction of one of the sensor axes is common to all four orientations.

Reference [29] proposes an activity recognition scheme invariant to sensor orientation and position, based on tri-axial accelerometers. Orientation invariance is achieved by calibration movements to estimate the sensor orientation. With sensor units fixed to the body, the subject performs two static postures for a few seconds. Then, the axes of a new coordinate frame are determined by using Gram-Schmidt ortho-normalization applied to the average acceleration vectors corresponding to the calibration postures.

In some studies [30,31], the sensor unit is allowed to be placed at an incorrect position on the same body part but its orientation is assumed to remain fixed throughout the activity, which is not realistic. Reference [30] claims that the sensor unit orientation can be estimated without much effort in most cases, which is not always true according to the results obtained in the literature [32,33].

In our earlier work [34,35,36], we have proposed two different approaches to transform and make time-domain sensor data invariant to the orientations at which the sensor units are fixed to the body. The first approach is a heuristic transformation where geometrical features invariant to the sensor unit orientation are extracted from the sensor data and used in the classification process [34,35], analogous to a method proposed by [37] for gait analysis. In the second approach, sensor sequences are represented with respect to three principal axes that are calculated using singular value decomposition (SVD) [34,36]. In both approaches, the transformed sequences are mathematically proven to be invariant to sensor unit orientations. Unlike most of the other studies that investigate orientation invariance, the proposed heuristic and SVD-based methods are compared with the fixed orientation case and shown to decrease the accuracy by 15.5% and 7.6%, respectively, on average, over five publicly available datasets, four state-of-the-art classifiers, and two cross-validation techniques. It is also shown in [34] that randomly oriented sensor units degrade the accuracy by 21.2% when the untransformed sensor data are used for classification. In this article, as explained in Section 4.3, we use a wider set of classifiers and leave-one-subject-out (L1O) cross-validation technique for better generalizability [38], in which case our newly proposed method achieves a noticeably higher accuracy than our previously proposed methods [34,35,36].

### 2.4. Discussion

The activity recognition methods in most of the existing studies are not generic and the results are neither consistent nor comparable because they use different datasets and sensor configurations. Furthermore, in most studies, the proposed orientation-invariant methods are not evaluated comparatively including the case with fixed sensor unit orientations. These methods either impose a major restriction on the possible sensor unit orientations or the types of body movements, which prevents them from being used in a wide range of applications such as health, state, and activity monitoring of elderly or disabled people. The aim of our study is to propose a novel orientation-invariant transformation and to comparatively and fairly observe its impact on the activity recognition accuracy based on the same dataset. To this end, we execute the activity recognition scheme with and without applying our transformation in the pre-processing stage for comparison between fixed and variable sensor unit orientations. We also implement the existing orientation invariance methods to compare them with ours. Furthermore, we artificially rotate the sensor data to observe the effects of incorrectly oriented sensor units on the standard activity recognition system that is originally designed for fixed sensor unit orientations.

## 3. Proposed Methodology to Achieve Invariance to Sensor Unit Orientation

To achieve orientation invariance with wearable motion sensor units in activity recognition, we propose to transform the acquired sensor data such that they become invariant to the orientations at which the sensor units are worn on the body. To transform the data, we first estimate the orientation of each sensor unit with respect to the Earth frame as a function of time. Unlike most existing studies, we consider a continuum of sensor orientations.

### 3.1. Estimation of Sensor Orientation

**Figure 3:**
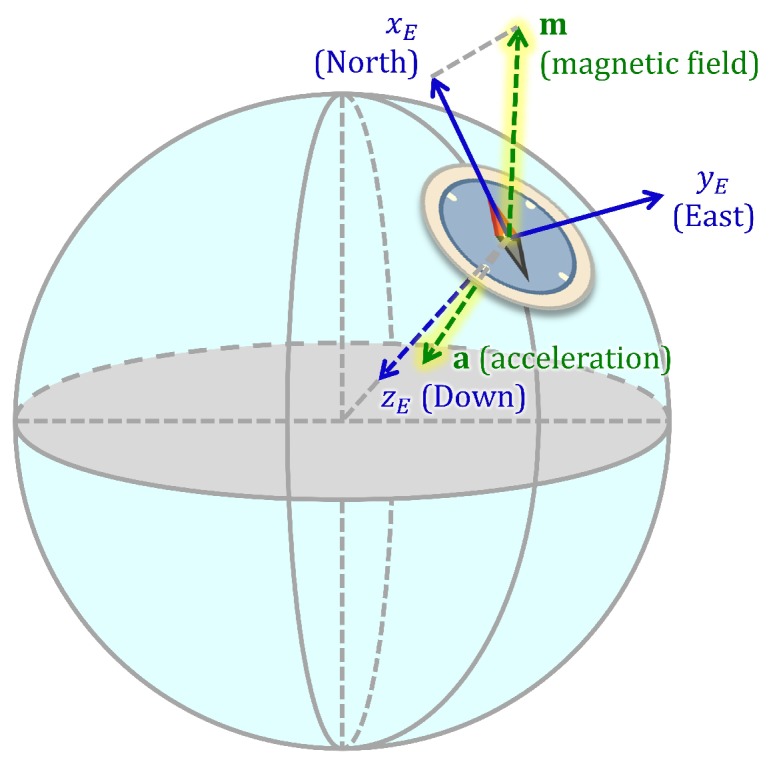
The Earth frame illustrated on an Earth model with the acquired reference vectors.

We define the Earth’s coordinate frame *E* such that the Earth’s *z* axis, $z_{E}$, points downwards and the Earth’s *x* axis, $x_{E}$, points in the direction of the component of the Earth’s magnetic field that is perpendicular to the *z* axis, which is roughly the North direction, as illustrated in Figure 3. The Earth frame is also called the North-East-Down frame [39].

**Figure 4:**
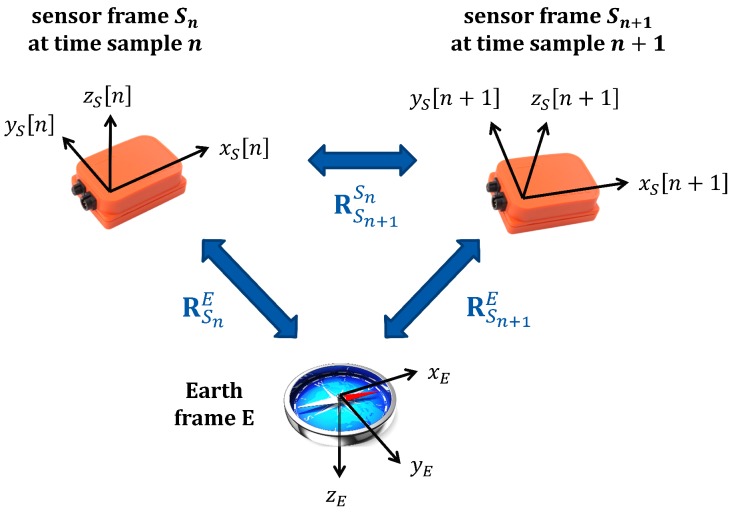
The Earth and the sensor coordinate frames at two consecutive time samples with the rotational transformations relating them.

Let $S_{n}$ be the rotating sensor frame at time sample *n*. Estimating the sensor unit orientation involves calculating a 3×3 rotational transformation matrix $R_{S_{n}}^{E}$ that describes the sensor frame $S_{n}$ with respect to the Earth frame *E* at each time sample *n*. The Earth frame and the sensor frame at consecutive time samples *n* and n+1 are depicted in Figure 4 together with the rotation matrices relating these coordinate frames. We adopt the orientation estimation method in [40], which is explained in the Appendix A. The short-term orientation estimate is calculated by integrating the angular rate acquired by the gyroscope. For the long-term orientation estimation, Gauss-Newton method is used to minimize a cost function which decreases as the acceleration vector points downwards in the Earth frame and as the horizontal component of the magnetic field vector is aligned with the North direction of the Earth frame. Then, the short- and long-term orientation estimates are combined through weighted averaging [40].

### 3.2. Sensor Signals with Respect to the Earth Frame

The tri-axial data acquired on the x,y, and *z* axes of each sensor in the sensor coordinate frame $S_{n}$ naturally depend on the orientations of the sensor units. Our approach is based on transforming the acquired data from the sensor frame to the Earth frame.

Let $v^{S}\left[n\right]={\left(v_{x}^{S}\left[n\right],v_{y}^{S}\left[n\right],v_{z}^{S}\left[n\right]\right)}^{T}$ be the data vector in $R^{3}$ acquired from the x,y,z axes of a tri-axial sensor at time sample *n*. To represent $v^{S}\left[n\right]$ with respect to the Earth frame, we pre-multiply it by the estimated sensor unit orientation at that time sample, which is the rotation matrix relating the $S_{n}$ frame to the *E* frame:

(1) $$v^{E}\left[n\right]=R_{S_{n}}^{E}\;v^{S}\left[n\right]$$

The components of the vector $v^{E}\left[n\right]={\left(v_{x}^{E}\left[n\right],v_{y}^{E}\left[n\right],v_{z}^{E}\left[n\right]\right)}^{T}$ are represented with respect to the $x_{E},y_{E},z_{E}$ axes of the Earth frame and are invariant to the sensor orientation.

### 3.3. Differential Sensor Rotations with Respect to the Earth Frame

In addition to the data transformed to the Earth frame, we propose to incorporate the information contained in the change in the sensor unit orientation over time. While the sensor units can be placed at arbitrary orientations, we require that during data acquisition their orientations remain fixed with respect to the body part they are placed on. In other words, the sensor units need to be firmly attached to the body and are not allowed to rotate freely during the motion. However, this restriction is only necessary in the short term over one time segment (5 s in this study). Under this restriction, the rotational motion of the body parts on which the sensor units are worn can be extracted from the acquired data correctly regardless of the initial orientations of the units.

Note that we can easily calculate the sensor unit orientation $R_{S_{n+1}}^{S_{n}}$ at time sample n+1 relative to the sensor orientation at time sample *n* as

(2) $$C_{n}≜R_{S_{n+1}}^{S_{n}}=R_{E}^{S_{n}}\;R_{S_{n+1}}^{E}={\left(R_{S_{n}}^{E}\right)}^{-1}\;R_{S_{n+1}}^{E}$$

for each *n* as shown in Figure 4. The matrix $C_{n}$ is not invariant to sensor orientation because it represents the orientation of frame $S_{n+1}$ with respect to $S_{n}$ and depends on the orientation at which the sensor unit is fixed to the body. To observe this, let us assume that the sensor unit is placed at a different arbitrary orientation; that is, the sensor unit is rotated by an arbitrary rotation matrix P that is constant over time. Then, the acquired data are ${\tilde{v}}^{S}\left[n\right]=P^{-1}\;v^{S}\left[n\right]$ for all *n*, represented with respect to the new sensor orientation ${\tilde{S}}_{n}$, and the sensor unit orientation with respect to the Earth is estimated as ${\tilde{R}}_{S_{n}}^{E}=R_{S_{n}}^{E}\;P$ for all *n*. Note that the original rotation matrix is post-multiplied by P because P describes a rotational transformation with respect to the sensor frame, not the Earth frame [41]. For the new sensor unit orientation, the rotation of the sensor unit between time samples *n* and n+1 can be calculated as

(3) $$\begin{array}{c} \begin{array}{cc} {\tilde{C}}_{n} & ={\tilde{R}}_{S_{n+1}}^{S_{n}}\\ & ={\tilde{R}}_{E}^{S_{n}}\;{\tilde{R}}_{S_{n+1}}^{E}\\ & ={\left({\tilde{R}}_{S_{n}}^{E}\right)}^{-1}\;{\tilde{R}}_{S_{n+1}}^{E}\\ & ={\left(R_{S_{n}}^{E}\;P\right)}^{-1}\;\left(R_{S_{n+1}}^{E}\;P\right)\\ & =P^{-1}\;{\left(R_{S_{n}}^{E}\right)}^{-1}\;R_{S_{n+1}}^{E}\;P\\ & =P^{-1}\;R_{E}^{S_{n}}\;R_{S_{n+1}}^{E}\;P\\ & =P^{-1}\;R_{S_{n+1}}^{S_{n}}\;P\\ & =P^{-1}\;C_{n}\;P \end{array} \end{array}$$

Since ${\tilde{C}}_{n}\neq C_{n}$ in general, $C_{n}$ is *not* invariant to sensor orientation.

We can make the rotational transformation $C_{n}$ invariant to sensor unit orientation by representing it in the Earth frame. Hence, we transform $C_{n}$ from the sensor frame $S_{n}$ to the Earth frame *E* by using a similarity transformation [42]:

(4) $$D_{n}={\left(R_{E}^{S_{n}}\right)}^{-1}C_{n}\left(R_{E}^{S_{n}}\right)=R_{S_{n}}^{E}\;R_{S_{n+1}}^{S_{n}}\;R_{E}^{S_{n}}=R_{S_{n+1}}^{E}\;R_{E}^{S_{n}}$$

We call this transformation $D_{n}$ *differential sensor rotation with respect to the Earth frame*.

It is straightforward to show that $D_{n}$ is invariant to sensor orientation. Using a constant arbitrary rotation matrix P that relates the original and modified sensor orientations as before, we have:

(5) $$\begin{array}{c} \begin{array}{cc} {\tilde{D}}_{n} & ={\tilde{R}}_{S_{n+1}}^{E}\;{\tilde{R}}_{E}^{S_{n}}\\ & ={\tilde{R}}_{S_{n+1}}^{E}{\left({\tilde{R}}_{S_{n}}^{E}\right)}^{-1}\\ & =\left(R_{S_{n+1}}^{E}\;P\right){\left(R_{S_{n}}^{E}\;P\right)}^{-1}\\ & =R_{S_{n+1}}^{E}\;\underset{I_{3\times 3}}{\underset{︸}{P\;P^{-1}}}{\left(R_{S_{n}}^{E}\right)}^{-1}\\ & =R_{S_{n+1}}^{E}\;R_{E}^{S_{n}}\\ & =D_{n} \end{array} \end{array}$$

Thus, we observe that the differential rotation ${\tilde{D}}_{n}$ with respect to the Earth frame, calculated based on the rotated data, is the same as the one calculated based on the original data $\left(D_{n}\right)$.

## 4. Comparative Evaluation of Proposed and Existing Methodology on Orientation Invariance for Activity Recognition

### 4.1. Dataset

**Figure 5:**
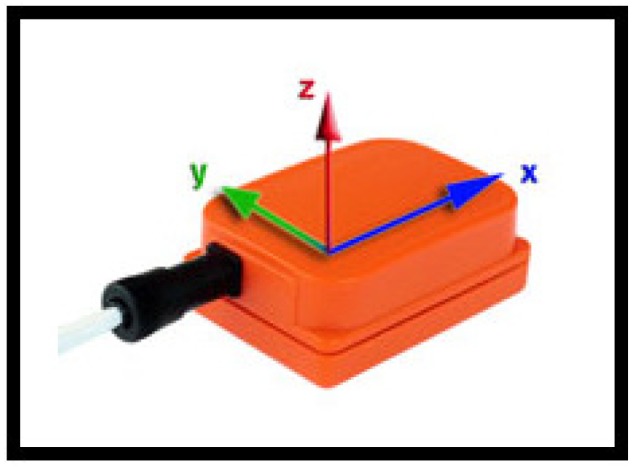
The Xsens MTx unit [44].

**Figure 6:**
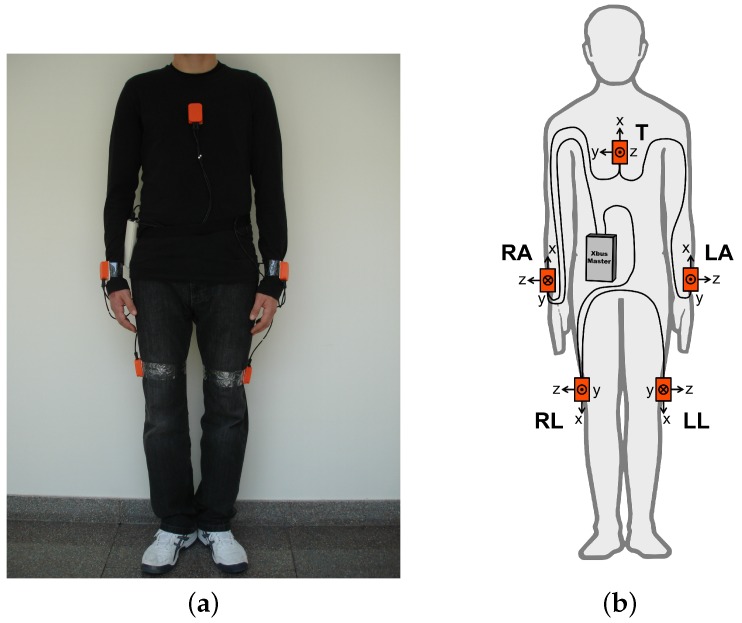
(**a**) Positioning of the MTx units on the body; (**b**) connection diagram of the units (the body drawing in the figure is from http://www.clker.com/clipart-male-figure-outline.html; the cables, Xbus Master, and sensor units were added by the authors).

To demonstrate our methodology, we use the publicly available daily and sports activities dataset acquired by our research group earlier [43]. To acquire the dataset, each subject wore five Xsens MTx sensor units [44] (see Figure 5), each unit containing three tri-axial devices: an accelerometer, a gyroscope, and a magnetometer. The sensor units are placed on the chest, on both wrists, and on the outer sides of both knees, as shown in Figure 6. Nineteen activities are performed by eight subjects. For each activity performed by each subject, there are 45 (=5units×9sensors) time-domain sequences of 5 min duration, sampled at 25 Hz, and consisting of 7500 time samples each. The dataset comprises the following activities:

Sitting (A${}_{1}$), standing (A${}_{2}$), lying on back and on right side (A${}_{3}$ and A${}_{4}$), ascending and descending stairs (A${}_{5}$ and A${}_{6}$), standing still in an elevator (A${}_{7}$), moving around in an elevator (A${}_{8}$), walking in a parking lot (A${}_{9}$), walking on a treadmill in flat and $15^{\circ }$ inclined positions at a speed of 4km/h (A${}_{10}$ and A${}_{11}$), running on a treadmill at a speed of 8km/h (A${}_{12}$), exercising on a stepper (A${}_{13}$), exercising on a cross trainer (A${}_{14}$), cycling on an exercise bike in horizontal and vertical positions (A${}_{15}$ and A${}_{16}$), rowing (A${}_{17}$), jumping (A${}_{18}$), and playing basketball (A${}_{19}$).

The activities can be broadly grouped into two: In stationary activities (A${}_{1}$–A${}_{4}$), the subject stays still without moving significantly, whereas non-stationary activities (A${}_{5}$–A${}_{19}$) are associated with some kind of motion.

### 4.2. Description of the Proposed and Existing Methodology on Orientation Invariance

In the pre-processing stage, seven data transformation techniques are considered to observe the effects of different sensor orientations on the accuracy and the improvement obtained with the existing and the proposed orientation-invariant transformations:

- **Reference:** Data are not transformed and the sensor units are assumed to maintain their fixed positions and orientations during the whole motion. This corresponds to the standard activity recognition scheme, as in [45,46,47].
- **Random rotation:** This case is considered to assess the accuracy of the standard activity recognition scheme (without any orientation-invariant transformation) when the sensor units are oriented randomly at their fixed positions. Instead of recording a new dataset with random sensor orientations, we randomly rotate the original data to make a fair comparison with the reference case. For this purpose, we randomly generate a rotational transformation:
  (6) $$P=\left[\begin{array}{ccc} 1 & 0 & 0\\ 0 & \cos\theta & -\sin\theta \\ 0 & \sin\theta & \cos\theta \end{array}\right]\left[\begin{array}{ccc} \cos\phi & 0 & \sin\phi \\ 0 & 1 & 0\\ -\sin\phi & 0 & \cos\phi \end{array}\right]\left[\begin{array}{ccc} \cos\psi & -\sin\psi & 0\\ \sin\psi & \cos\psi & 0\\ 0 & 0 & 1 \end{array}\right]$$
  where yaw, pitch, roll angles θ,ϕ,ψ are independent and uniformly distributed in the interval [−π,π) radians. For each time segment of each sensor unit (see Section 4.3 for segmentation), we generate a different P matrix and pre-multiply each of the three tri-axial sequences of that unit by the random rotation matrix corresponding to that segment of the unit: $\tilde{v}\left[n\right]=P\;v^{S}\left[n\right]$. In this way, we simulate the situation where each sensor unit is placed at a possibly different random orientation in each time segment.
- **Euclidean norm method:** The Euclidean norm of the x,y,z components of the sensor sequences are taken at each time sample and used instead of using the original tri-axial sequences. As reviewed in Section 2, this technique has been used in activity recognition to achieve sensor orientation invariance [10,11,12] or as an additional feature as in [13,14,15,16,48,49].
- **Sequences along and perpendicular to the gravity vector:** In this method, the acceleration sequence in each time segment is averaged over time to approximately calculate the direction of the gravity vector. Then, for each sensor type, the sensor sequence’s amplitude in this direction and the magnitude that is perpendicular to this direction are taken. This method has been used in [17,18,19] to achieve orientation invariance.
- **SVD-based transformation:** Sensory data are represented with respect to three principal axes that are calculated by SVD [34,36]. The transformation is applied to each time segment of each sensor unit separately so that sensor units are allowed to be placed at different orientations in each segment.

To calculate the orientation-invariant transformations in the remaining two methods, we estimate the orientation $R_{S_{n}}^{E}$ of each of the five sensor units as a function of time sample *n* as explained in the Appendix A. For the algorithm to reach steady state rapidly, we append to the acquired signal a prefix signal of duration 1 s that consists of zero angular rate, a constant acceleration, and a constant magnetic field that are the same as the measurements at the first time sample.

**Figure 7:**
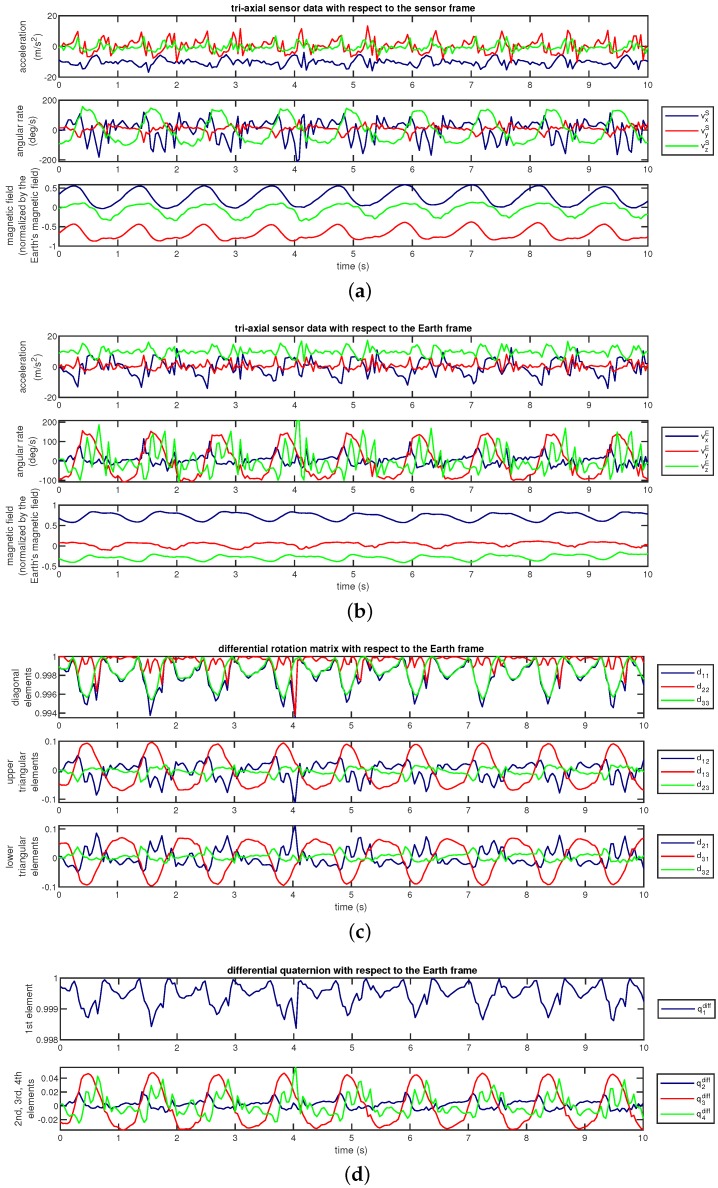
Original and orientation-invariant sequences from a walking activity plotted over time. (**a**) Original sensor sequences; (**b**) sensor sequences; elements of (**c**) the differential rotation matrix and (**d**) the differential quaternion. Sequences in (**b**–**d**) are represented in the Earth frame and are invariant to sensor orientation.

- **Sensor sequences with respect to the Earth frame:** We transform the sensor sequences into the Earth frame using the estimated sensor orientations, as described by Equation (1). This method has been used in [22] to achieve invariance to sensor orientation in activity recognition.
  As an example, Figure 7a shows the accelerometer, gyroscope, and magnetometer data $\left(v^{S}\left[n\right]\right)$ acquired during activity A${}_{10}$ and Figure 7b shows the same sequences transformed into the Earth frame. We observe that the magnetic field with respect to the Earth frame does not significantly vary over time because the Earth’s magnetic field is nearly constant in the Earth frame provided that there are no external magnetic sources in the vicinity of the sensor unit.
- **Proposed method: sensor sequences and differential quaternions, both with respect to the Earth frame:** We calculate the differential rotation matrix $D_{n}$ with respect to the Earth frame for each sensor unit at each time sample *n*, as explained in Section 3.3. This rotation matrix representation is quite redundant because it has nine elements while any 3D rotation can be represented by only three angles. Since the representation by three angles has a singularity problem, we represent the differential rotation $D_{n}$ compactly by a four-element quaternion $q_{n}^{\mathrm{diff}}$ as
  (7) $$q_{n}^{\mathrm{diff}}=\left[\begin{array}{c} q_{1}^{\mathrm{diff}}\\ q_{2}^{\mathrm{diff}}\\ q_{3}^{\mathrm{diff}}\\ q_{4}^{\mathrm{diff}} \end{array}\right]=\left[\begin{array}{c} \frac{\sqrt{1+d_{11}+d_{22}+d_{33}}}{2}\\ \frac{d_{32}-d_{23}}{4\sqrt{1+d_{11}+d_{22}+d_{33}}}\\ \frac{d_{13}-d_{31}}{4\sqrt{1+d_{11}+d_{22}+d_{33}}}\\ \frac{d_{21}-d_{12}}{4\sqrt{1+d_{11}+d_{22}+d_{33}}} \end{array}\right]$$
  where $d_{ij}\;\;\;\left(i,j=1,2,3\right)$ are the elements of $D_{n}$ [50]. The vector $q_{n}^{\mathrm{diff}}$ is called *differential quaternion with respect to the Earth frame* (the dependence of the elements of $q_{n}^{\mathrm{diff}}$ and $D_{n}$ on *n* has been dropped from the notation for simplicity). In the classification process, we use each element of $q_{n}^{\mathrm{diff}}$ as a function of *n*, as well as the sensor sequences with respect to the Earth frame. Hence, there are four time sequences for the differential quaternion in addition to the three axes each of accelerometer, gyroscope, and magnetometer data for each of the five sensor units. Therefore, the transformed data comprises (4+3+3+3)sequences×5sensorunits=65 sequences in total.
  We have observed that the joint use of the sensor sequences and differential quaternions, both with respect to the Earth frame, achieves the highest activity recognition accuracy compared to the other combinations. Representing rotational transformations by rotation matrices instead of quaternions degrades the accuracy. Omitting magnetometer sequences with respect to the Earth frame causes a slight reduction in the accuracy. Activity recognition results of the various different approaches that we have implemented are not presented in this article for brevity, and can be found in [51].
  Figure 7c shows the nine elements of the differential rotation matrix $D_{n}$ with respect to the Earth frame over time, which are calculated based on the sensor data shown in Figure 7a. Figure 7d shows the elements of the differential quaternion $q_{n}^{\mathrm{diff}}$ as a function of *n*. The almost periodic nature of the sensor sequences (Figure 7a) is preserved in $D_{n}$ and $q_{n}^{\mathrm{diff}}$ (Figure 7c,d). The differential rotation is calculated between two consecutive time samples that are only a fraction of a second apart, hence the amplitudes of the elements of $D_{n}$ and $q_{n}^{\mathrm{diff}}$ do not vary much. Since differential rotations involve small rotation angles (close to 0${}^{\circ }$), the $D_{n}$ matrices are close to the 3×3 identity matrix $\left(I_{3\times 3}\right)$ because they can be expressed as the product of three rotation matrices as in Equation (6) where each of the basic rotation matrices (as well as their product) is close to $I_{3\times 3}$ because of the small angles. Hence, the diagonal elements which are close to one and the upper- and lower-diagonal elements which are close to zero are plotted separately in Figure 7c for better visualization. When $D_{n}$ is close to $I_{3\times 3}$, the $q_{n}^{\mathrm{diff}}$ vectors calculated by using Equation (7) are close to ${\left(1,0,0,0\right)}^{T}$, as observed in Figure 7d.

### 4.3. Activity Recognition and Classifiers

A procedure similar to that in [34,45] is followed for activity recognition. The sensor sequences are divided into 9120 (=60featurevectors×19activities×8subjects) non-overlapping segments of 5 s duration each and transformed according to one of the seven approaches described in Section 4.2. Then, statistical features are extracted for each segment of each axis of each sensor type. The following features are calculated: minimum, maximum, mean, variance, skewness, kurtosis, 10 coefficients of the autocorrelation sequence (autocorrelation sequence for the lag values of 5,10,…,45,50 samples is used), and the five largest discrete Fourier transform (DFT) peaks with the corresponding frequencies (the separation between any two peaks in the DFT sequence is taken to be at least 11 samples), resulting in a total of 26 features per segment of each axis. For the reference approach that does not involve any transformation, there are 5sensorunits×9axes×26featuresperaxis=1170 features that are stacked to form a 1170-element feature vector for each segment. The number of axes as well as the number of features vary depending on the transformation technique; however, the total number of feature vectors is fixed (9120). For instance, in the Euclidean norm, there is a three-fold decrease in the number of axes and hence in the number of features. The features are normalized to the interval [0,1] over all the feature vectors for each subject.

**Figure 8:**
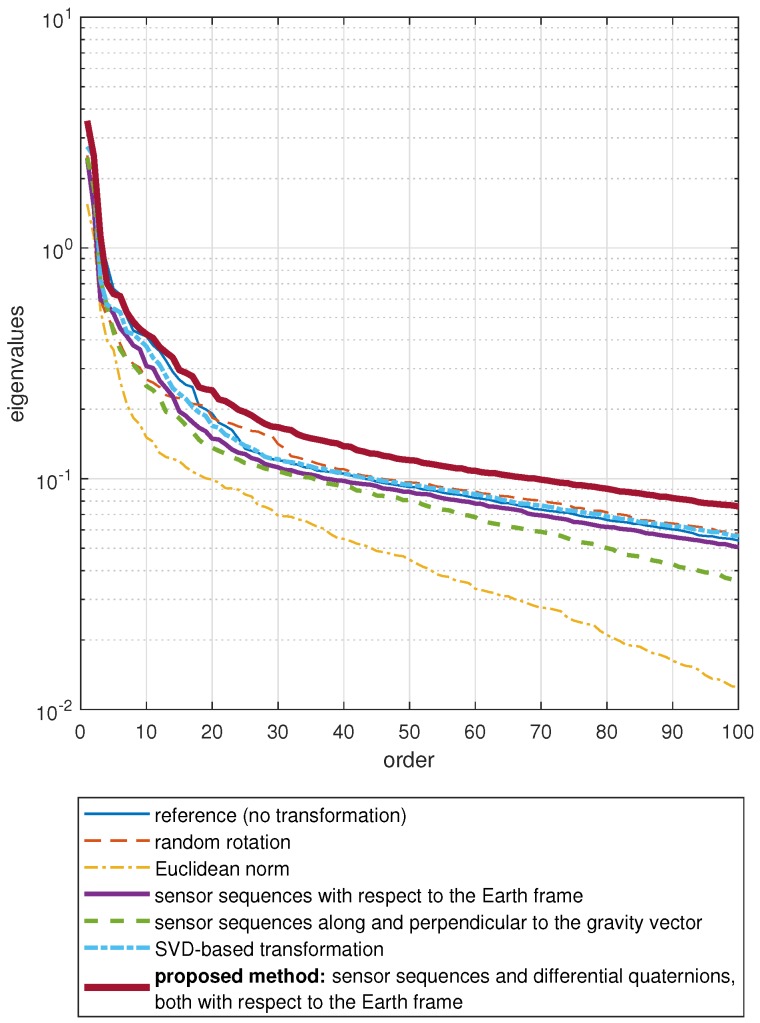
The first 100 eigenvalues of the covariance matrix of the feature vectors sorted in descending order, calculated based on the features extracted from the data transformed according to the seven approaches.

The number of features is reduced through PCA, which is a linear and orthogonal transformation where the transformed features are sorted to have variances in descending order [52]. This allows one to consider only a certain number of features that exhibit the largest variances to reduce the dimensionality. Thus, for each approach, the eigenvalues of the covariance matrix of the feature vectors are calculated, sorted in descending order, and plotted in Figure 8. Using the first 30 eigenvalues appears to be suitable for most of the approaches; hence, we reduce the dimensionality down to F=30.

We perform activity classification with seven state-of-the-art classifiers that are briefly described below.

- **Support Vector Machines (SVM):** The feature space is nonlinearly mapped to a higher-dimensional space by using a kernel function and divided into regions by hyperplanes. In this study, the kernel is selected to be a Gaussian radial basis function $f_{\mathrm{RBF}}\left(x,y\right)=e^{{-\gamma ∥x-y∥}^{2}}$ with parameter γ because it can perform at least as accurately as the linear kernel if the parameters of the SVM are optimized [53]. To extend the binary SVM to more than two classes, a binary SVM classifier is trained for each class pair, and the decision is made according to the classifier with the highest confidence level [54]. The penalty parameter *C* (see Equation (1) in [55]) and the kernel parameter γ are jointly optimized over all the data transformation techniques by performing a two-level grid search. The optimal parameter values in the coarse grid $\left(C,\;\gamma \right)\in \left\{10^{-5},\;10^{-3},\;10^{-1},\;\ldots ,\;10^{15}\right\}\times \left\{10^{-15},\;10^{-13},\;10^{-11},\;\ldots ,\;10^{3}\right\}$ are obtained as $\left(C^{*},\;\gamma^{*}\right)=\left(10^{1},\;10^{-1}\right)$. Then, a finer grid is constructed around $\left(C^{*},\;\gamma^{*}\right)$ as $\left(C,\;\gamma \right)\in 100\;P\times P$ with $P=\left\{0.01,\;0.05,\;0.1,\;0.2,\;0.3,\;0.4,\;0.5,\;0.7,\;1,\;3,\;5\right\}$ and the optimal parameter values found by searching the fine grid, $\left(C^{**},\;\gamma^{**}\right)=\left(5,\;0.1\right)$, are used in SVM throughout this study. The SVM classifier is implemented by using the MATLAB toolbox LibSVM [56].
- **Artificial Neural Networks (ANN):** We use three layers of neurons, where each neuron has a sigmoid output function [57]. The number of neurons in the first (input) and the third (output) layers are as many as the reduced number of features, *F*, and the number of classes, *K*, respectively. The number of neurons in the second (or hidden) layer is selected as the integer nearest to the average of $\frac{\log\left(2K\right)}{\log2}$ and 2K−1, with the former expression corresponding to the optimistic case where the hyperplanes intersect at different positions and the latter corresponding to the pessimistic case where the hyperplanes are parallel to each other. The weights of the linear combination in each neuron are initialized randomly in the interval [0,0.2] and during the training phase, they are updated by the back-propagation algorithm [58]. The learning rate is selected as 0.3. The algorithm is terminated when the amount of error reduction (if any) compared to the average of the last 10 epochs is less than 0.01. The ANN has a scalar output for each class. A given test feature vector is fed to the input and the class corresponding to the largest output is selected.
- **Bayesian Decision Making (BDM):** In the training phase, a multi-variate Gaussian distribution with an arbitrary covariance matrix is fitted to the training feature vectors of each class. Based on maximum likelihood estimation, the mean vector is estimated as the arithmetic mean of the feature vectors and the covariance matrix is estimated as the sample covariance matrix for each class. In the test phase, for each class, the test vector’s conditional probability given that it is associated with that class is calculated. The class that has the maximum conditional probability is selected according to the maximum a posteriori decision rule [52,57].
- **Linear Discriminant Classifier (LDC):** This classifier is the same as BDM except that the average of the covariance matrices individually calculated for each class is used for all of the classes. Since the Gaussian distributions fitted to the different classes have different mean vectors but the same covariance matrix in this case, the classes have identical probability density functions centered at different points in the feature space. Hence, the classes are linearly separated from each other, and the decision boundaries in the feature space are hyperplanes [57].
- ***k*-Nearest Neighbor (*k*-NN):** The training phase consists only of storing the training vectors with their class labels. In the classification phase, the class corresponding to the majority of the *k* training vectors that are closest to the test vector in terms of the Euclidean distance is selected [57]. The parameter *k* is chosen as k=7 because it is suitable among the *k* values ranging from 1 to 30.
- **Random Forest (RF):** A random forest classifier is a combination of multiple decision trees [59]. In the training phase, each decision tree is trained by randomly and independently sampling the training data. Normalized information gain is used as the splitting criterion at each node. In the classification phase, the decisions of the trees are combined by using majority voting. The number of decision trees is selected as 100 because we have observed that using a larger number of trees does not significantly improve the accuracy while increasing the computational cost considerably.
- **Orthogonal Matching Pursuit (OMP):** The training phase consists of only storing the training vectors with their class labels. In the classification phase, each test vector is represented as a linear combination of a very small portion of the training vectors with a bounded error, which is called the sparse representation. The vectors in the representation are selected iteratively by using the OMP algorithm [60] where an additional training vector is selected at each iteration. The algorithm terminates when the desired representation error level is reached, which is selected to be $10^{-3}$. Then, a residual for each class is calculated as the representation error when the test vector is represented as a linear combination of the training vectors of only that class, and the class with the minimum residual error is selected.

To determine the accuracies of the classifiers, L1O cross-validation technique is used [57]. In this type of cross validation, feature vectors of a given subject are left out while training the classifier with the remaining subjects’ feature vectors. The left out subject’s feature vectors are then used for testing (classification). This process is repeated for each subject. Thus, in our implementation, the dataset is partitioned into eight and there are 1140 feature vectors in each partition. L1O is highly affected by the variation in the data across the subjects, and hence, is more challenging than subject-unaware cross-validation techniques such as repeated random sub-sampling or multi-fold cross validation [61].

### 4.4. Comparative Evaluation Results

**Figure 9:**
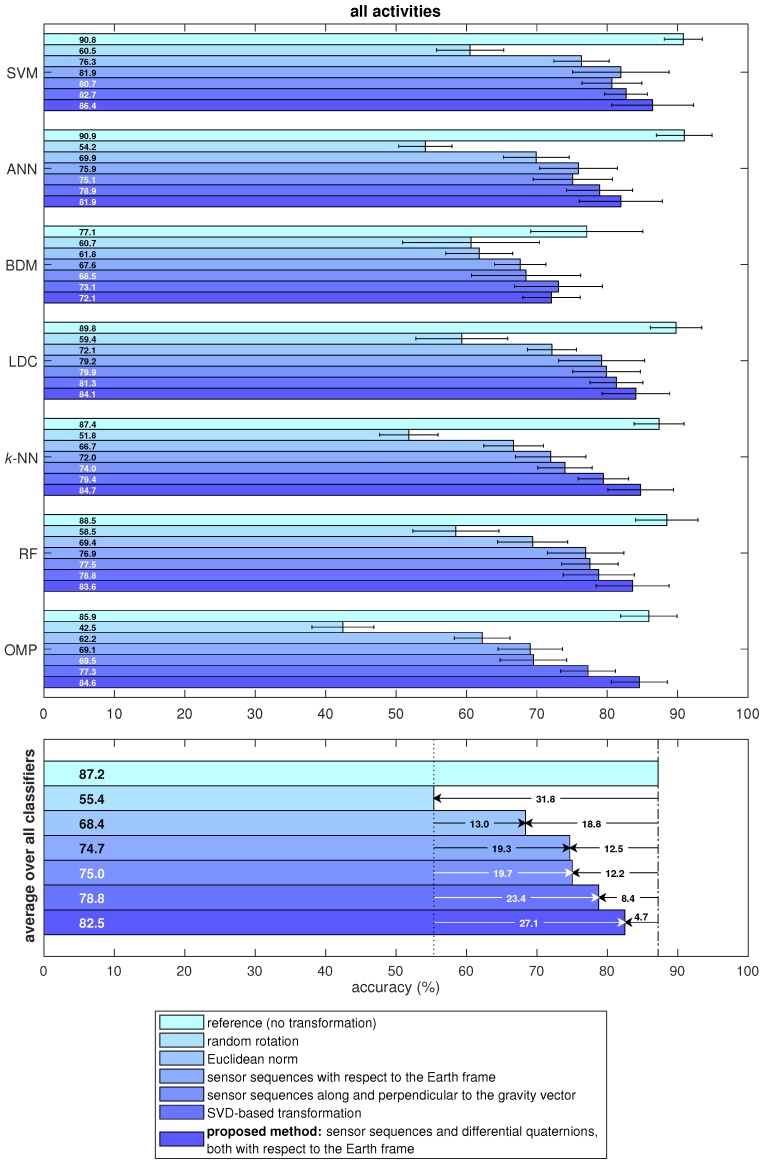
Activity recognition performance for all the data transformation techniques and classifiers over all activities. The lengths of the bars represent the accuracies and the thin horizontal sticks indicate plus/minus one standard deviation over the cross-validation iterations.

The activity recognition performance of the different data transformation techniques and classifiers is shown in Figure 9. In the figure, the lengths of the bars correspond to the classification accuracies and the thin horizontal sticks indicate plus/minus one standard deviation about the accuracies averaged over the cross-validation iterations.

In the lower part of Figure 9, the accuracy values averaged over the seven classifiers are also provided for each approach and compared with the reference case, as well as with the proposed method. Referring to this part of the figure, the standard system that we take as reference, with fixed sensor orientations, provides an average accuracy of 87.2%. When the sensor units are randomly oriented, the accuracy drops by 31.8% on average with respect to the standard reference case. This shows that the standard system is not robust to incorrectly or differently oriented sensors. The existing methods for orientation invariance result in a more acceptable accuracy reduction compared to the reference case: The accuracy drop is 18.8% when the Euclidean norms of the tri-axial sensor sequences are taken, 12.5% when the sensor sequences are transformed to the Earth frame, 12.2% when the sensor sequences are represented along and perpendicular to the gravity vector, and 8.4% when the SVD-based transformation is applied.

Our approach that uses the sensor sequences together with differential quaternions, both with respect to the Earth frame, achieves an average accuracy of 82.5% over all activities with an average accuracy drop of only 4.7% compared to the reference case. Such a decrease in the accuracy is expected when the sensor units are allowed to be placed freely at arbitrary orientations because this flexibility entails the removal of fundamental information such as the direction of the gravity vector measured by the accelerometers and the direction of the Earth’s magnetic field detected by the magnetometers. Hence, the average accuracy drop of 4.7% is considered to be acceptable when such information related to the sensor unit orientations is removed inevitably.

In the lower part of Figure 9, we also provide the improvement achieved by each method compared to the random rotation case which corresponds to the standard system using random sensor orientations. The method that we newly propose in this article performs the best among all the methods considered in this study when the sensor units are allowed to be placed at arbitrary orientations.

The activity recognition accuracy highly depends on the classifier. According to Figure 9, in almost all cases, the SVM classifier performs the best among the seven classifiers compared. SVM outperforms the other classifiers especially in approaches targeted to achieve orientation invariance where the classification problem is more challenging. The robustness of SVM in such non-ideal conditions is consistent with other studies [13,46]. Besides the SVM classifier, ANN and LDC also obtain high classification accuracy. Although reference [22] states that *k*-NN has been shown to perform remarkably well in activity recognition, it is not the most accurate classifier that we have identified.

To observe the recognition rates of the individual activities, a confusion matrix associated with the SVM classifier is provided in Table 1 for the proposed method. It is apparent that the proposed transformation highly misclassifies the stationary activities A${}_{1}$–A${}_{4}$. These activities contain stationary postures, namely, sitting, standing, and two types of lying, which are misclassified probably because we remove the information about sensor orientation from the data. In particular, activity A${}_{1}$ (sitting) is mostly misclassified and confused with activities A${}_{3}$ (lying on back side) and A${}_{7}$ (standing still in an elevator). The remaining stationary activities are also misclassified as A${}_{7}$. Among the 15 non-stationary activities, activities A${}_{10}$ and A${}_{11}$ (walking on a treadmill in flat and $15^{\circ }$ inclined position, respectively) are confused with each other because of the similarity between the body movements in the two activities. Other misclassifications occur between activity pairs that have similarities such as A${}_{7}$/A${}_{8}$, A${}_{8}$/A${}_{7}$, A${}_{2}$/A${}_{8}$, A${}_{18}$/A${}_{6}$, and A${}_{13}$/A${}_{9}$, although rarely. Activities A${}_{12}$ (running on a treadmill at a speed of 8 km/h) and A${}_{17}$ (rowing) are perfectly classified by SVM for the proposed method, probably because they are associated with unique body movements and do not resemble any of the other activities.

**Figure 10:**
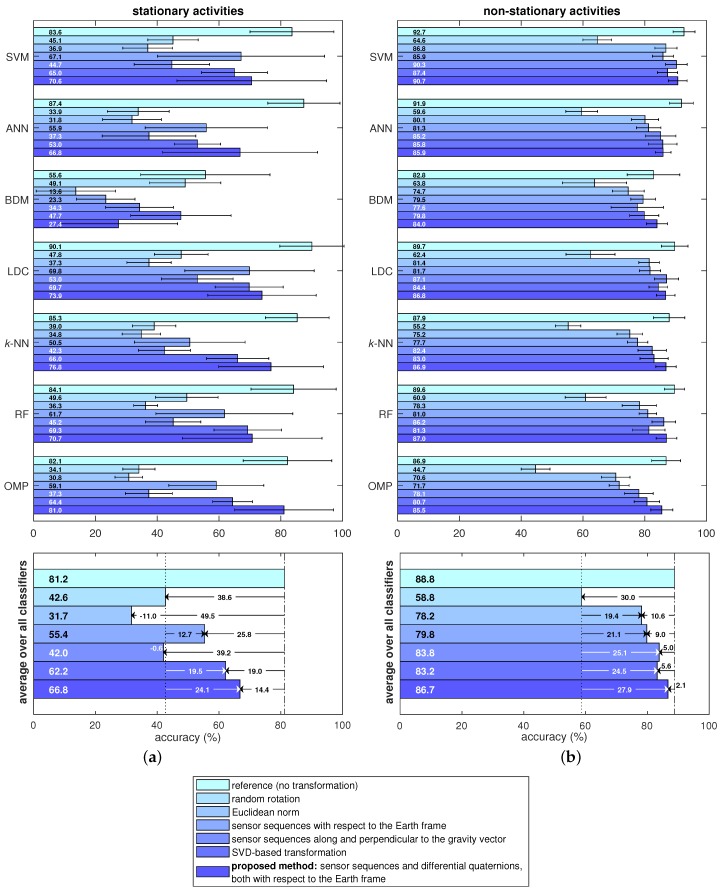
Activity recognition performance for all the data transformation techniques and classifiers for (**a**) stationary and (**b**) non-stationary activities. The lengths of the bars represent the accuracies and the thin horizontal sticks indicate plus/minus one standard deviation over the cross-validation iterations.

We present the classification performance separately for stationary and non-stationary activities in Figure 10. For each classifier and each approach, we calculate the accuracy values by averaging out the accuracies of the stationary activities (A${}_{1}$–A${}_{4}$) and non-stationary activities (A${}_{5}$–A${}_{19}$).

For stationary activities (see Figure 10a), an average accuracy of 81.2% is obtained for fixed sensor orientations. When the sensor units are oriented randomly, the average accuracy drops to 42.6%. The existing orientation-invariant methods exhibit accuracies between 31.7% and 62.2%, some of them being higher and some being lower than the accuracy for random rotation. The Euclidean norm method performs particularly poorly in this case. The proposed method achieves an average accuracy of 66.8%, which is considerably higher than random rotation and all the existing orientation-invariant transformations. Although two of the existing transformations provide some improvement compared to the random rotation case, their accuracies are much lower than the standard reference system. Hence, removing the orientation information from the data makes it particularly difficult to classify stationary activities.

For non-stationary activities (see Figure 10b), the accuracy decreases from 88.8% to 58.8% on average when the sensor units are placed randomly and no transformation is applied. The existing orientation-invariant methods obtain accuracies ranging from 78.2% to 83.2%, which are comparable to the reference case with fixed sensor orientations. The method we propose obtains an average accuracy of 86.7%, which is higher than all the existing methods and only 2.1% lower than the reference case. This shows that when the sensor units are fixed to the body at arbitrary orientations, the proposed method can classify non-stationary activities with a performance similar to that of fixed sensor unit orientations. In the last two rows of the confusion matrix provided in Table 1, the average accuracy of the stationary activities (A${}_{1}$–A${}_{4}$) and non-stationary activities (A${}_{5}$–A${}_{19}$) are provided separately for the proposed method, again using the SVM classifier.

Referring to Figure 10a, we observe that the recognition rate of stationary activities highly depends on the classifier. On average, the best classifier is LDC, probably because the recognition of stationary activities is quite challenging and the LDC classifier separates the classes from each other linearly and smoothly in the feature space. For the proposed method, the OMP classifier performs much better than the remaining six classifiers. On the other hand, for non-stationary activities (see Figure 10b), the classifiers obtain comparable accuracy values, unlike the case for stationary activities. In this case, SVM is the most accurate classifier, both on average and for the proposed method.

### 4.5. Run Time Analysis

The average run times of the data transformation techniques per one 5-s time segment are provided in Table 2. All the processing in this work was performed on a laptop with a quad-core Intel^®^ Core^TM^ i7-4720HQ processor at 2.6–3.6 GHz and 16 GB of RAM running 64-bit MATLAB^®^ R2017b. The proposed method has an average run time of about 61 ms per 5-s time segment and can be executed in near real time since the run time is much shorter than the duration of the time segment.

The run times of the classifiers are presented in Table 3 for each of the seven data transformation techniques. Table 3a contains the total run times of the classifiers for an average cross-validation iteration, including the training phase and classification of all the test feature vectors. We observe that *k*-NN, LDC, and BDM are much faster than the other classifiers for all of the data transformation techniques. Table 3b contains the average training times of the classifiers for a single cross-validation iteration. The *k*-NN and OMP classifiers only store the training feature vectors in the training phase; therefore, their training time is negligible. Among the remaining classifiers, training of BDM is the fastest. Table 3c contains the average classification time of a single test feature vector, extracted from a segment of 5 s duration. ANN and LDC are about an order of magnitude faster than the others in classification. The classification time of OMP is the largest. Note that, because of programming overheads, the total classification times provided in Table 3a are greater than the sum of the training and classification times (Table 3b,c, respectively) multiplied by 1140 (the number of feature vectors per L1O iteration).

This study is a proof-of-concept for a comparative analysis of the accuracies and run times of the proposed and existing methods as well as state-of-the-art classifiers. Therefore, we have implemented them as well as the remaining parts of the activity recognition framework on a laptop computer rather than on a mobile platform.

Given that the data transformation techniques and most of the classifiers have been implemented in MATLAB in this study, it is possible to further improve the efficiency of the algorithms by programming them in other languages such as C++, by implementing them on an FPGA platform, or by embedding the algorithms in wearable hardware. As such, our methodology can be handled by the limited resources of wearable systems. Alternatively, transmitting the data acquired from wearable devices wirelessly to a cloud server would allow performing the activity recognition in the cloud [14,62]. Despite the latency issues that will arise in this case, this approach would provide additional flexibility and enable the applications of wearables to further benefit from the proposed methodology and the advantages of cloud computing.

## 5. Discussion

Overall, the recognition rates of non-stationary activities are considerably better than those of stationary ones for all the approaches considered in this study. This is because in non-stationary activities, the activity type is encoded in the body motion whereas in stationary activities, since there is no significant body motion, the removal of sensor orientation information to achieve orientation invariance has a major impact on the accuracy. The classification of stationary activities is a more challenging problem and it is clear that sensor unit orientations provide essential information for this purpose.

The direction of the gravity vector measured by the accelerometer and the direction of the magnetic field vector determined by the magnetometer provide essential information about the orientation of the sensor unit. When the sensor sequences are represented with respect to the Earth frame to achieve orientation invariance, this information is lost because the gravity and the magnetic field of the Earth are roughly in the fixed $z_{E}$ and $x_{E}$ directions of the Earth frame, respectively. Hence, in our proposed method, we incorporate the change in the sensor unit orientation over time by calculating differential quaternions with respect to the Earth, which represent the rotation between consecutive time samples invariantly to the sensor unit orientation. The use of differential quaternions increases the accuracy considerably because they effectively represent the rotational motion of the sensor unit related to the activities. When the rotational transformation is represented with respect to the Earth frame, it is invariant to sensor unit orientation, as desired.

For all the methods compared in this study, we use the same dataset which was acquired by placing the sensor units on the body at fixed orientations. This enables us to make a fair comparison between all of the seven approaches considered in this work. In the random rotation case, we rotate the data arbitrarily for each time segment and each sensor unit; hence, we obtain new data that simulate random sensor orientations and match exactly the same level of difficulty of the original data except for the rotational difference. In the last five approaches that correspond to orientation-invariant methods, it is mathematically guaranteed that the transformed data are exactly invariant to sensor orientations; hence, they can be directly compared with the reference and random rotation cases. Had we recorded an additional dataset with different sensor unit orientations, we would not be able to fairly compare the accuracies obtained with the two datasets because it is not possible to guarantee the same level of difficulty in activity recognition in different experiments. This fact can be observed even within the current dataset from the non-negligible standard deviations in the activity recognition accuracy over the cross-validation iterations (see Figure 9 and Figure 10). This shows that the variation among the subjects is significant, as also observed in [38].

## 6. Conclusions and Future Work

We have demonstrated that the standard activity recognition paradigm cannot handle incorrectly or differently oriented sensors when the position remains fixed. To overcome this problem, we have proposed a transformation that we apply on the sensor data at the pre-processing stage to increase the robustness of the system to errors in the orientations at which the sensor units are worn on the body. The method we have proposed extracts the activity-related information from the sensor sequences while removing the information associated with the absolute sensor unit orientations. This way, we ensure that the transformed sequences do not depend on the absolute sensor unit orientations. The transformed sequences have the same form as the original sequences except the number of axes, which enables us to apply this method in the pre-processing stage of any system that can handle multi-axial data, including systems that directly use time-domain data in its raw form as well as those that use extracted features. We have shown that our method significantly reduces the accuracy degradation caused by incorrect/different sensor unit orientations. The proposed method performs substantially better than the existing methods developed specifically for this problem and achieves nearly the same accuracy level as the fixed orientation case for non-stationary activities. The transformation we propose can be computed in a time much shorter than the duration of one segment of the data, therefore, it can be efficiently implemented and used in near real time.

The next step of this research may involve calculating the differential quaternions with respect to the Earth over a wider time window rather than over only two consecutive time samples, which may improve robustness against high-frequency noise. The transformation proposed here can be used in other wearable sensing applications such as detecting and classifying falls and automated evaluation of physical therapy exercises. By transforming the sensor data at the pre-processing stage, orientation invariance can be achieved without the need to modify the rest of the system. Position invariance can also be investigated to allow the sensor units to be interchanged and/or placed at different positions on the body. The two can be combined to develop activity recognition systems that are invariant to both the position and orientation of the sensor units. 

## Figures and Tables

**Table 1:** Confusion matrix of the SVM classifier for the proposed method over all activities.

Estimated Labels							True Labels																Total
		A_1_	A_2_	A_3_	A_4_		A_5_	A_6_	A_7_	A_8_	A_9_	A_10_	A_11_	A_12_	A_13_	A_14_	A_15_	A_16_	A_17_	A_18_	A_19_		
A${}_{1}$		286	1	68	20		0	0	8	0	0	0	0	0	0	0	0	0	0	0	0		383
A${}_{2}$		0	330	0	0		0	0	26	1	0	0	0	0	0	0	0	0	0	0	0		357
A${}_{3}$		81	0	372	0		0	0	0	0	0	0	0	0	0	0	0	0	0	0	0		453
A${}_{4}$		1	0	0	367		0	0	0	0	0	0	0	0	0	0	0	0	0	0	0		368
A${}_{5}$		0	0	0	0		477	0	0	2	11	0	12	0	0	0	0	0	0	0	1		503
A${}_{6}$		0	0	0	0		0	453	2	5	0	0	0	0	6	0	0	0	0	42	0		508
A${}_{7}$		97	102	33	83		0	0	354	61	0	0	0	0	0	0	0	0	0	0	0		730
A${}_{8}$		15	47	6	10		1	27	90	409	1	0	1	0	9	2	2	0	0	0	8		628
A${}_{9}$		0	0	0	0		2	0	0	1	416	19	4	0	36	0	0	1	0	0	0		479
A${}_{10}$		0	0	0	0		0	0	0	0	13	354	84	0	0	0	0	0	0	0	0		451
A${}_{11}$		0	0	0	0		0	0	0	0	38	105	374	0	11	0	0	0	0	0	0		528
A${}_{12}$		0	0	0	0		0	0	0	0	0	0	0	480	0	0	0	0	0	0	0		480
A${}_{13}$		0	0	0	0		0	0	0	1	1	2	5	0	399	7	0	0	0	0	1		416
A${}_{14}$		0	0	0	0		0	0	0	0	0	0	0	0	19	471	0	0	0	0	0		490
A${}_{15}$		0	0	0	0		0	0	0	0	0	0	0	0	0	0	478	1	0	0	0		479
A${}_{16}$		0	0	0	0		0	0	0	0	0	0	0	0	0	0	0	477	0	0	0		477
A${}_{17}$		0	0	1	0		0	0	0	0	0	0	0	0	0	0	0	0	480	0	1		482
A${}_{18}$		0	0	0	0		0	0	0	0	0	0	0	0	0	0	0	1	0	438	0		439
A${}_{19}$		0	0	0	0		0	0	0	0	0	0	0	0	0	0	0	0	0	0	469		469
total		480	480	480	480		480	480	480	480	480	480	480	480	480	480	480	480	480	480	480		9120
**accuracy (%)**		59.6	68.8	77.5	76.5		99.4	94.4	73.8	85.2	86.7	73.8	77.9	100.0	83.1	98.1	99.6	99.4	100.0	91.3	97.7		**86.5 (overall)**
		**70.6**					**90.7**																
		(for stationary activities)					(for non-stationary activities)																

**Table 2:** Average run times of the data transformation techniques per 5-s time segment.

Data Transformation Technique	Run Time (ms)
Euclidean norm	0.69
sensor sequences with respect to the Earth frame	56.25
sensor sequences along and perpendicular to the gravity vector	1.09
SVD-based transformation	8.94
**proposed method:** sensor sequences and differential	61.08
quaternions, both with respect to the Earth frame	

**Table 3:** (**a**) Total run time (including training and classification of all test feature vectors) and (**b**) training time in an average L1O iteration; (**c**) average classification time of a single test feature vector.

	Classifier	Reference (No Transformation)	Random Rotation	Euclidean Norm	Sensor Sequences with Respect to the Earth Frame	Sensor Sequences Along and Perpendicular to the Gravity Vector	SVD-Based Transformation	Proposed Method: Sensor Sequences and Differential Quaternions, Both with Respect to the Earth Frame
**(a)** **total run time** **(s)**	SVM	6.42	14.20	7.22	11.71	8.19	6.24	10.05
	ANN	7.37	8.49	8.54	6.58	12.04	7.91	6.14
	BDM	1.67	1.61	1.59	1.55	2.12	1.48	1.69
	LDC	1.10	0.87	0.84	1.52	0.84	0.93	1.51
	*k*-NN	0.24	0.12	0.12	0.21	0.19	0.12	0.22
	RF	16.81	22.51	26.40	24.34	19.05	19.71	23.98
	OMP	1018.27	798.90	92.32	99.41	96.48	75.18	114.68
**(b)** **training time** **(s)**	SVM	6.01	13.39	6.61	10.31	7.58	5.36	8.60
	ANN	7.35	8.47	8.52	6.57	12.01	7.89	6.12
	BDM	0.01	0.01	0.01	0.01	0.01	0.01	0.01
	LDC	0.33	0.23	0.22	0.38	0.22	0.26	0.33
	*k*-NN	–	–	–	–	–	–	–
	RF	15.20	20.90	24.11	21.75	17.45	17.87	21.25
	OMP	–	–	–	–	–	–	–
**(c)** **classification time** **(ms)**	SVM	0.26	0.60	0.42	0.39	0.40	0.24	0.31
	ANN	0.02	0.02	0.01	0.01	0.02	0.01	0.01
	BDM	1.46	1.41	1.39	1.35	1.85	1.29	1.47
	LDC	0.04	0.03	0.03	0.05	0.03	0.03	0.04
	*k*-NN	0.21	0.11	0.11	0.19	0.16	0.11	0.19
	RF	0.71	0.73	0.99	0.83	0.72	0.74	0.87
	OMP	892.55	700.17	80.55	86.38	84.20	65.43	99.69

## Abbreviations

SVM	Support Vector Machines
PCA	Principal Component Analysis
1-NN	One-Nearest-Neighbor
SVD	Singular Value Decomposition
DFT	Discrete Fourier Transformation
ANN	Artificial Neural Networks
BDM	Bayesian Decision Making
LDC	Linear Discriminant Classifier
*k*-NN	*k*-Nearest Neighbor
RF	Random Forest
OMP	Orthogonal Matching Pursuit
L1O	Leave-One-Subject-Out

## Appendix A. Sensor Unit Orientation Estimation

The orientation estimation method in [40] combines orientation estimates based on two sources of information. The first estimate is obtained simply by integrating the gyroscope angular rate measurements. This estimate is accurate in the short term but drifts in the long term. The second relies on the direction of the gravity vector measured by the accelerometer and the magnetic field of the Earth detected by the magnetometer in the long term. For the long-term estimation, the Gauss-Newton method [40] is used to solve a minimization problem where the cost function decreases as the acquired acceleration vector is aligned with the gravity vector and as the acquired magnetic field vector is aligned with the magnetic North of the Earth. The short- and long-term estimates are combined through weighted averaging [40].

In the orientation estimation algorithm, we relate the sensor and the Earth frames by a quaternion ${\hat{q}}_{n}={\left(q_{1},\;q_{2},\;q_{3},\;q_{4}\right)}^{T}$ corresponding to the rotation matrix ${\hat{R}}_{E}^{S_{n}}={\left({\hat{R}}_{S_{n}}^{E}\right)}^{-1}$ for all *n* as follows [50]:

(A1) $${\hat{R}}_{E}^{S_{n}}=\left[\begin{array}{ccc} q_{1}^{2}+q_{2}^{2}-q_{3}^{2}-q_{4}^{2} & 2\left(q_{2}q_{3}-q_{1}q_{4}\right) & 2\left(q_{1}q_{2}+q_{2}q_{4}\right)\\ 2\left(q_{2}q_{3}+q_{1}q_{4}\right) & q_{1}^{2}-q_{2}^{2}+q_{3}^{2}-q_{4}^{2} & 2\left(q_{3}q_{4}-q_{1}q_{2}\right)\\ 2\left(q_{2}q_{4}-q_{1}q_{3}\right) & 2\left(q_{1}q_{2}+q_{3}q_{4}\right) & q_{1}^{2}-q_{2}^{2}-q_{3}^{2}+q_{4}^{2} \end{array}\right]$$

The short- and long-term orientation estimates are denoted by ${\hat{q}}_{n,\;\mathrm{ST}}$ and ${\hat{q}}_{n,\;\mathrm{LT}}$ and the overall estimate is denoted by ${\hat{q}}_{n}$.

The short-term estimate of the sensor quaternion ${\hat{q}}_{n,\;\mathrm{ST}}$ at time sample *n* based on the overall estimate ${\hat{q}}_{n-1}$ at the previous time sample is given by:

(A2) $${\hat{q}}_{n,\;\mathrm{ST}}={\hat{q}}_{n-1}+\Delta t\left(\frac{1}{2}{\hat{q}}_{n-1}⊗\omega^{S}\left[n\right]\right)$$

where $\omega^{S}\left[n\right]={\left(0,\;\omega_{x}^{S}\left[n\right],\;\omega_{y}^{S}\left[n\right],\;\omega_{z}^{S}\left[n\right]\right)}^{T}$ is an augmented vector consisting of zero and the angular rate vector acquired by the gyroscope at time sample *n* [40] and Δt is the sampling interval. Note that the equation involves feedback because ${\hat{q}}_{n,\;\mathrm{ST}}$ is calculated based on ${\hat{q}}_{n-1}$.

For the long-term estimation, let $a^{S}\left[n\right]$ and $m^{S}\left[n\right]$ be the acceleration and the magnetic field vectors, respectively, represented in the sensor frame and normalized by their magnitudes. To align $a^{S}\left[n\right]$ with the $z_{E}$ axis of the Earth frame, we represent it in the Earth frame as $a^{E}\left[n\right]=q_{n}⊗a^{S}\left[n\right]⊗q_{n}^{*}$, and solve the following minimization problem [40]:

(A3) $${\hat{q}}_{n,\;\mathrm{LT}-1}=\underset{q_{n}}{\arg\;\min}\;f_{1}\;\left(q_{n},\;a^{S}\left[n\right]\right)\;\mathrm{where}\;f_{1}\;\left(q_{n},\;a^{S}\left[n\right]\right)=∥{\left(0,\;0,\;1\right)}^{T}-q_{n}⊗a^{S}\left[n\right]⊗q_{n}^{*}∥$$

where ∥·∥ denotes the Euclidean norm and ⊗ denotes the quaternion product operator.

We represent the magnetic field vector $m^{S}\left[n\right]$ as $m^{E}\left[n\right]=q_{n}⊗m^{S}\left[n\right]⊗q_{n}^{*}$ in the Earth frame and allow it to have only a vertical component along the $z_{E}$ direction and a horizontal component along the $x_{E}$ direction. Hence, we align $m^{E}\left[n\right]$ with the magnetic reference vector defined as $m_{0}\left[n\right]≜{\left(\sqrt{{\left(m_{x}^{E}\left[n\right]\right)}^{2}+{\left(m_{y}^{E}\left[n\right]\right)}^{2}},\;0,\;m_{z}^{E}\left[n\right]\right)}^{T}$ in the Earth frame by solving the following minimization problem [40]:

(A4) $${\hat{q}}_{n,\;\mathrm{LT}-2}=\underset{q_{n}}{\arg\;\min}\;f_{2}\;\left(q_{n},\;m^{S}\left[n\right]\right)\;\mathrm{where}\;f_{2}\;\left(q_{n},\;m^{S}\left[n\right]\right)=∥m_{0}\left[n\right]-q_{n}⊗m^{S}\left[n\right]⊗q_{n}^{*}∥$$

To simultaneously align the acceleration and magnetic field vectors, we combine the minimization problems defined in Equations (A3) and (A4) into one and solve the following joint minimization problem:

(A5) $${\hat{q}}_{n,\;\mathrm{LT}}=\underset{q_{n}}{\arg\;\min}\;f\left(q_{n},\;a^{S}\left[n\right],\;m^{S}\left[n\right]\right)$$

where the combined objective function is

(A6) $$f\left(q_{n},\;a^{S}\left[n\right],\;m^{S}\left[n\right]\right)=f_{1}^{2}\;\left(q_{n},\;a^{S}\left[n\right]\right)+f_{2}^{2}\;\left(q_{n},\;m^{S}\left[n\right]\right)$$

We use the Gauss-Newton method to solve the problem defined in Equation (A5) iteratively [40]. The quaternion at iteration i+1 can be calculated based on the estimate at the *i*th iteration as follows:

(A7) $$q_{n,\;\mathrm{LT}}^{\left(i+1\right)}=q_{n,\;\mathrm{LT}}^{\left(i\right)}-{\left(J^{T}J\right)}^{-1}J^{T}\;f\left(q_{n,\;\mathrm{LT}}^{\left(i\right)},\;a^{S}\left[n\right],\;m^{S}\left[n\right]\right)$$

where J is the 6×4 Jacobian matrix of f with respect to the elements of $q_{n}^{\left(i\right)}$. This matrix is provided in closed form in [40].

Finally, the short- and long-term estimates are merged by using weighted averaging [40]:

(A8) $${\hat{q}}_{n}=K{\hat{q}}_{n,\;\mathrm{ST}}+\left(1-K\right){\hat{q}}_{n,\;\mathrm{LT}}$$

where the parameter K is selected as 0.98 as in [40]. The estimated quaternion ${\hat{q}}_{n}$ represents the rotation matrix ${\hat{R}}_{E}^{S_{n}}$ compactly, where we drop the hat notation $\left(\hat{\;\;}\right)$ in the main text for simplicity.
